# Supplementary material for: Arabidopsis SWR1-associated protein methyl-CpG-binding domain 9 is required for histone H2A.Z deposition
Source: Nat Commun. 2019 Jul 26;10:3352. doi: 10.1038/s41467-019-11291-w (PMC6659704; doi:10.1038/s41467-019-11291-w)
Supplement: Supplementary file 1 — Supplementary Information [file 41467_2019_11291_MOESM1_ESM.pdf]

Arabidopsis SWR1-associated protein methyl-CpG-binding domain 9 is required for histone H2A.Z deposition

Potok *et al.*

Supplementary Methods

ChIP-seq analysis

Fastq reads were aligned to the TAIR10 reference genome excluding chromosome chloroplast and mitochondria with Bowtie[1] using default settings and allowing only uniquely mapping reads. Duplicate reads were removed using SAMtools[2]. Pearson correlation between H2A.Z ChIP-seq replicates was generated with R using normalized (RPM) 1kb binned windows generated using biotoolbox application get_datasets.pl (https://github.com/tjparnell/biotoolbox). Normalized read coverage tracks were generated using the USeq package Sam2Useq application[3]. IGB genome browser was used to visualize the data and to generate snapshots[4]. H2A.Z enriched peaks vs corresponding H3 signal or between mutants for each replicate were determined by callpeak function in MACS2 (v2.1.1.)[5] with the following parameters, -g 1.3e8 –q 0.01 –extsize 200. MBD9, enriched gene were identified similarly as H2A.Z enriched peaks using wild-type Flag ChIP-seq as control. To obtain common H2A.Z enriched peaks between replicates, H2A.Z enriched peaks for each sample were intersected using the USeq package IntersectRegions[3] application with gap =0. Genes corresponding to enriched H2A.Z peaks and MBD9 occupied genes were identified by intersecting with TAIR10 UCSC gene table using the USeq package IntersectRegions application[3]. BioVenn application was used to generate the overlap between H2A.Z enriched genes[6]. ChIP-seq metaplots were generated using NGSPLOT (v4.02.48)[7] with moving window =5. Annotation of peak locations were carried out using the HOMER annotatepeaks function[8]. GO term enrichment was determined using AgriGO[9]. Clustering of H2A.Z profiles in wild-type over MBD9-occupied genes was generated with NGSPLOT (v4.02.48)[7] using *k*-means clustering, n=5, with maximum number of iterations =20 and number of random starts =30.

BS-seq analysis

For generating average methylation plots over MBD9-occupied genes and control set of regions, publicly available wild-type BS-seq data in seedlings was used and analyzed as follows. Trim_galore (http://www.bioinformatics.babraham.ac.uk/projects/trim_galore/) have been used to trim adapters after filtering low quality reads. BS-seq reads were aligned to TAIR10 reference genome by Bismark (v0.18.2)[10] with default settings. Reads with three or more consecutive CHH sites were considered as unconverted reads and have been filtered. DNA methylation levels were defined as #C/ (#C + #T). For meta plot of methylation data, up and down flanking 1000 bp sequences were divided into ten 100bp-bins respectively and MBD9 peaks regions were divided into 10 proportioned bins, then calculate average CG, CHG, and CHH methylation level at these bins.

RNA-seq analysis

Reads were aligned to TAIR10 using Tophat[11] by allowing up to two mismatches and mapping only to one location. FRPKM values and differential gene expression were analyzed using Cuffdiff[11] with default settings. BioVenn application was used to generate the overlap between differentially expressed genes[6]. Boxplots of expression were generated using R, unpaired two-samples Wilcoxon test was used to determine statistical significance between samples. GO term enrichment was determined using AgriGO[12]. To investigate the misregulation of transcription factors with differentially expressed genes in our mutants, transcriptional factor genes and families were obtained from the Arabidopsis transcription factors database (agris-knowledgebase.org) and intersected with upregulated and down-regulated genes in each mutants. The overlap of transcription factors up-regulated and down-regulated in each mutant was generated using BioVenn.

ATAC-seq analysis

ATAC-seq read adaptors were removed with trim_galore (http://www.bioinformatics.babraham.ac.uk/projects/trim_galore/) and then mapped to the *Arabidopsis* thaliana reference genome TAIR10 using Bowtie (-X 2000 -m 1)[1]. Chloroplast and mitochondrial DNA aligned reads were filtered out and duplicate reads were removed using SAMtools[2].

Defining H2A.Z gene classes

To classify genes according to H2A.Z profiles in wild-type, we used the same approach as described in Coleman-Derr, D. and Zilberman D., 2012[13]. Specifically, H2A.Z levels (RPKM) were obtained for TSS regions (TSS + 500bp) and gene body regions (between TSS +500bp and TES -500bp) for protein coding genes using biotoolbox application get_datasets.pl. We empirically determined that to best represent short genes less than 1500bp, H2A.Z levels were obtained from the entire coding region and the same value was assigned to both TSS and TES region. Next the regions were divided into three groups based on the levels of H2A.Z at gene body (values in log 2 RPKM, low < 3 (9612 genes), medium 3 to <4 (8467 genes), high > =4 (8805 genes)). The three categories were further equally divided into three groups representing low, medium, and high levels of TSS. The categories are designated as L – Low H2A.Z, M- Medium H2A.Z, and H- High H2A.Z for TSS (first letter), and gene body (second letter) as follows (LL, ML, HL, LM, MM, HM, LH, MH, and HH). In total, this analysis represents 26884 protein coding genes. The levels of H2A.Z, gene expression, and gene length are represented by boxplots generated in R for each category of genes. The overlap between each class of genes with H2A.Z and RNA-seq differential genes for each mutant was determined using BioVenn.

Supplementary Note

The following published dataset were used for the data analyses in this study:
H3K4me3 – 10 day seedlings – (accession number GSE49090, https://www.ncbi.nlm.nih.gov/geo/query/acc.cgi?acc=GSE49090)[14]
H3K27me3 – 14 day seedlings – (accession number GSE53620, https://www.ncbi.nlm.nih.gov/geo/query/acc.cgi?acc=GSE53620)[15]
DNA methylation - Col-0 seedling BS-seq data (accession number SRR520367, https://www.ncbi.nlm.nih.gov/sra/SRR520367)[16]

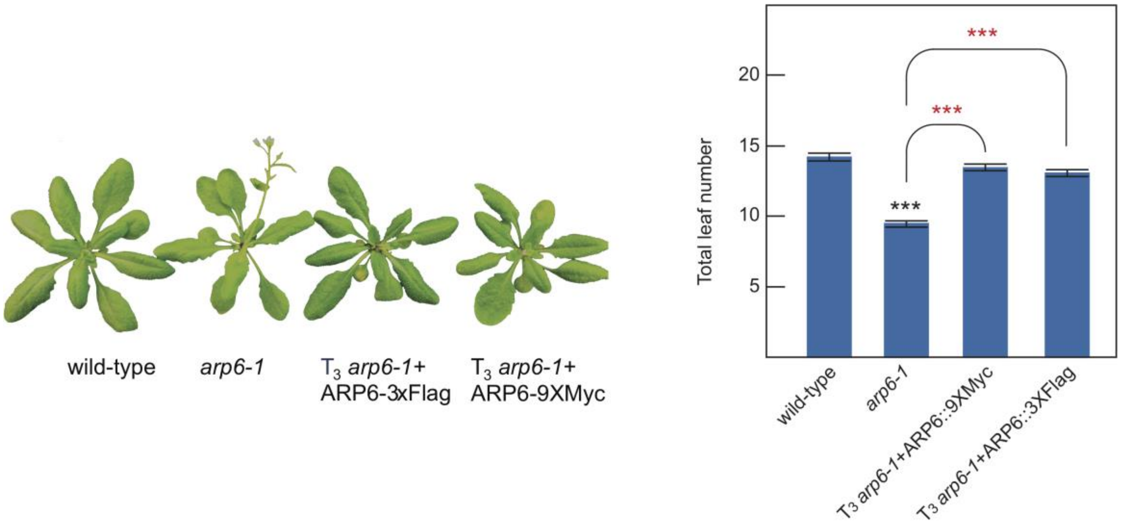

Supplementary Figure 1. **Complementation of the *arp6-1* phenotype.** Morphological phenotype of wild-type, *arp6-1*, T_3 *arp6-1+ARP6::3xFlag*, and T_3 *arp6-1+ARP6::9xMyc*. Plants were grown for 5 weeks under long-day conditions. Flowering time expressed as the total number of leaves produced by wild-type, *arp6-1*, T_3 *arp6-1+ARP6::3xFlag*, and T_3 *arp6-1+ARP6::9xMyc* from 16 plants ± standard deviations under the same conditions is also shown. Paired two-tailed Student's t-test was used to determine significance between wild-type and the mutant (black asterisks) or the transgenic lines (red asterisks); ns p-value > 0.05, * p-value <= 0.05, ** p-value <= 0.01, *** p-value <= 0.001. Source data is provided as Source Data file.

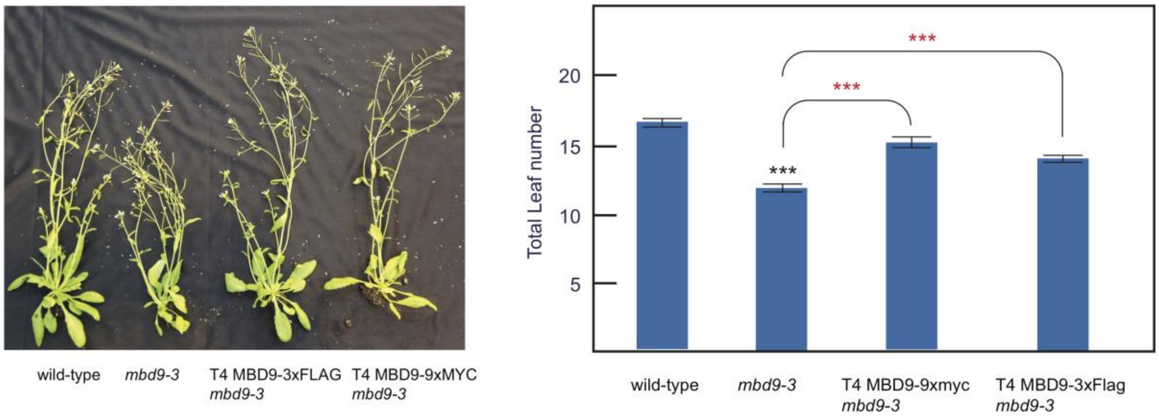

Supplementary Figure 2. Complementation of the *mbd9-3* phenotype. Morphological phenotype of wild-type, *mbd9-3*, T_4 *mbd9-3+MBD9::3xFlag*, and T_4 *mbd9-3+MBD9::9xMyc*. Plants were grown for 7 weeks under long-day conditions. Flowering time expressed as the total number of leaves produced by wild-type, *mbd9-3*, T_4 *mbd9-3+MBD9::3xFlag*, and T_4 *mbd9-3+MBD9::9xMyc* from 12 plants ± standard deviations at 5 weeks is shown. Paired two-tailed Student's t-test was used to determine significance between wild-type and the mutant (black asterisks) or the transgenic lines (red asterisks); ns p-value > 0.05, * p-value <= 0.05, ** p-value <= 0.01, *** p-value <= 0.001. Source data is provided as Source Data file.

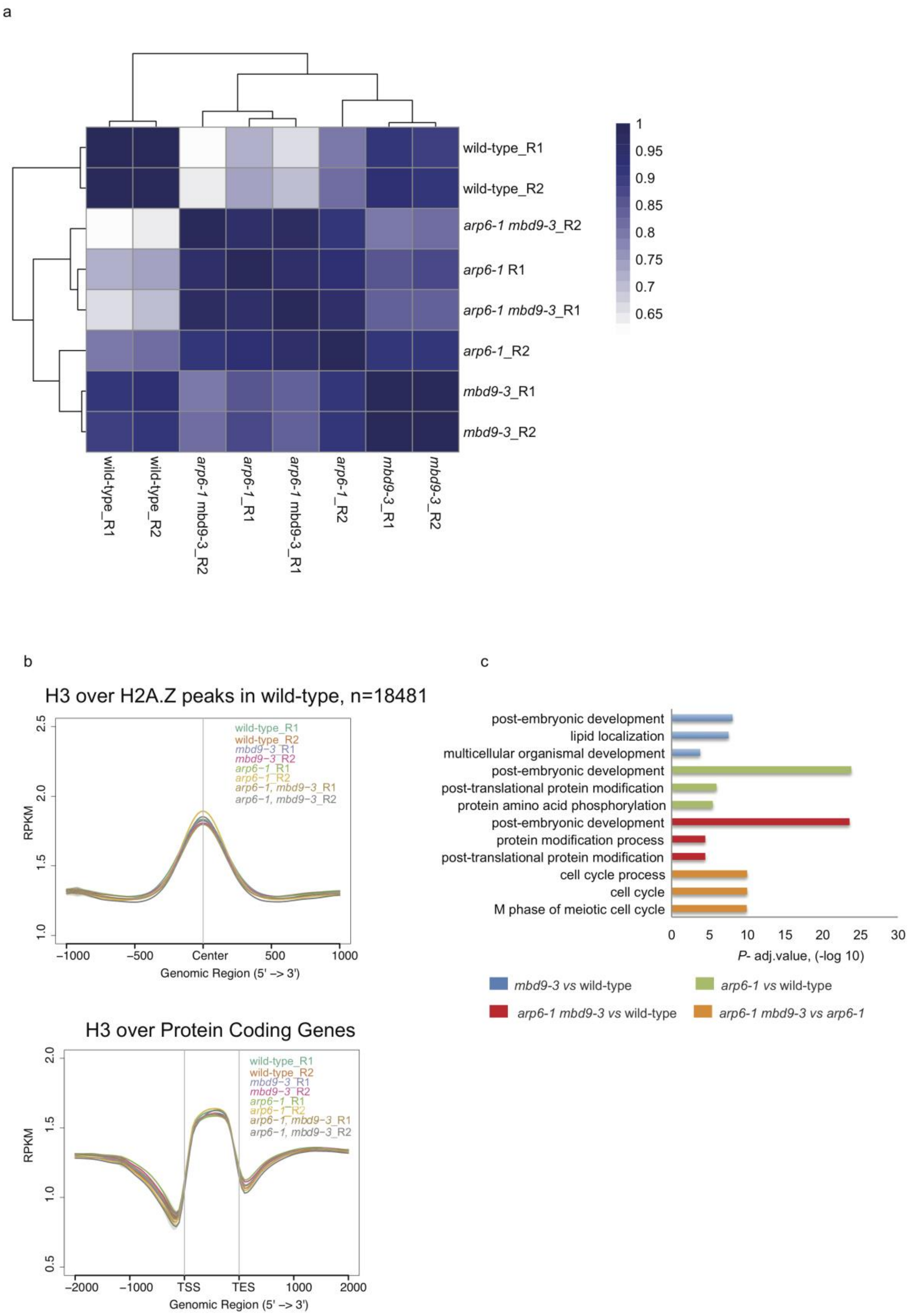
a
wild-type_R1
wild-type_R2
arp6-1 mbd9-3_R2
arp6-1 R1
arp6-1 mbd9-3_R1
arp6-1_R2
mbd9-3_R1
mbd9-3_R2
wild-type_R1
wild-type_R2
arp6-1 mbd9-3_R2
arp6-1_R1
arp6-1 mbd9-3_R1
arp6-1_R2
mbd9-3_R1
mbd9-3_R2
1
0.95
0.9
0.85
0.8
0.75
0.7
0.65
b
H3 over H2A.Z peaks in wild-type, n=18481
wild-type_R1
wild-type_R2
mbd9-3_R1
mbd9-3_R2
arp6-1_R1
arp6-1_R2
arp6-1, mbd9-3_R1
arp6-1, mbd9-3_R2
RPKM
1.0
1.5
2.0
2.5
−1000
−500
Center
500
1000
Genomic Region (5' -> 3')
H3 over Protein Coding Genes
wild-type_R1
wild-type_R2
mbd9-3_R1
mbd9-3_R2
arp6-1_R1
arp6-1_R2
arp6-1, mbd9-3_R1
arp6-1, mbd9-3_R2
RPKM
0.5
1.0
1.5
2.0
−2000
−1000
TSS
TES
1000
2000
Genomic Region (5' -> 3')
c
post-embryonic development
lipid localization
multicellular organismal development
post-embryonic development
post-translational protein modification
protein amino acid phosphorylation
post-embryonic development
protein modification process
post-translational protein modification
cell cycle process
cell cycle
M phase of meiotic cell cycle
0
5
10
15
20
25
30
P- adj.value, (-log 10)
mbd9-3 vs wild-type
arp6-1 vs wild-type
arp6-1 mbd9-3 vs wild-type
arp6-1 mbd9-3 vs arp6-1

Supplementary Figure 3. Correlation of H2A.Z ChIP-seq and profiles of H3 ChIP-seq. (a) Pearson correlation of normalized 1 kb binned H2A.Z ChIP-seq signal (RPM) for indicated replicates. **(b)** Distribution of normalized H3 ChIP-seq signal (RPKM) for each ChIP-seq replicate over H2A.Z common peaks in wild-type and protein-coding genes. **(c)** GO term analysis for H2A.Z-depleted genes (macs2 peak caller, q-value less than 0.01). *P*-adjusted value in –log(10) is shown for top three GO classes for the indicated mutants.

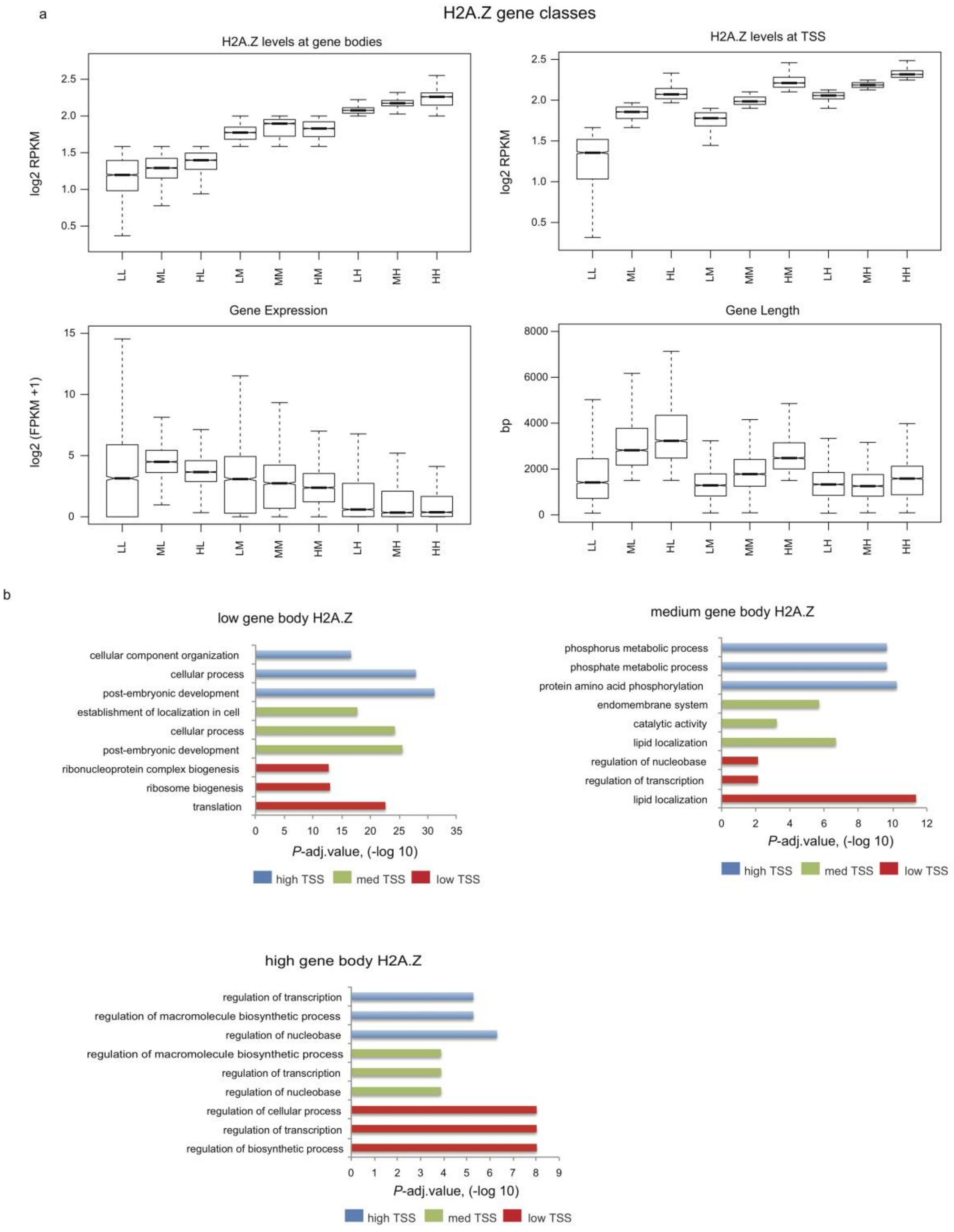

Supplementary Figure 4. Features of gene classes characterized according to their levels of H2A.Z at TSS and gene body. H2A.Z levels (RPKM) were calculated for TSS regions (TSS + 500 bp**)** and gene body regions **(**between TSS +500 bp and

TES -500 bp) for protein-coding genes. **(a)** Notched boxplots of H2A.Z levels (RPKM) at gene bodies and TSS from merged replicates of H2A.Z ChIP-seq in wild-type. Centre line indicates the median, upper and lower bounds represent the 75th and the 25th percentile respectively, whiskers indicate the minimum and the maximum, outliers are not plotted. Gene expression in $\log_2$ (FPKM+1) in wild-type and gene length in bp for each class. Labels H – high, M – medium, L- low, refer to levels of H2A.Z at TSS (first letter) and gene body (second letter). (b) GO term analysis for the nine classes of genes. *P*-adjusted value in –log(10) is shown for the top three GO classes for the indicated mutants.

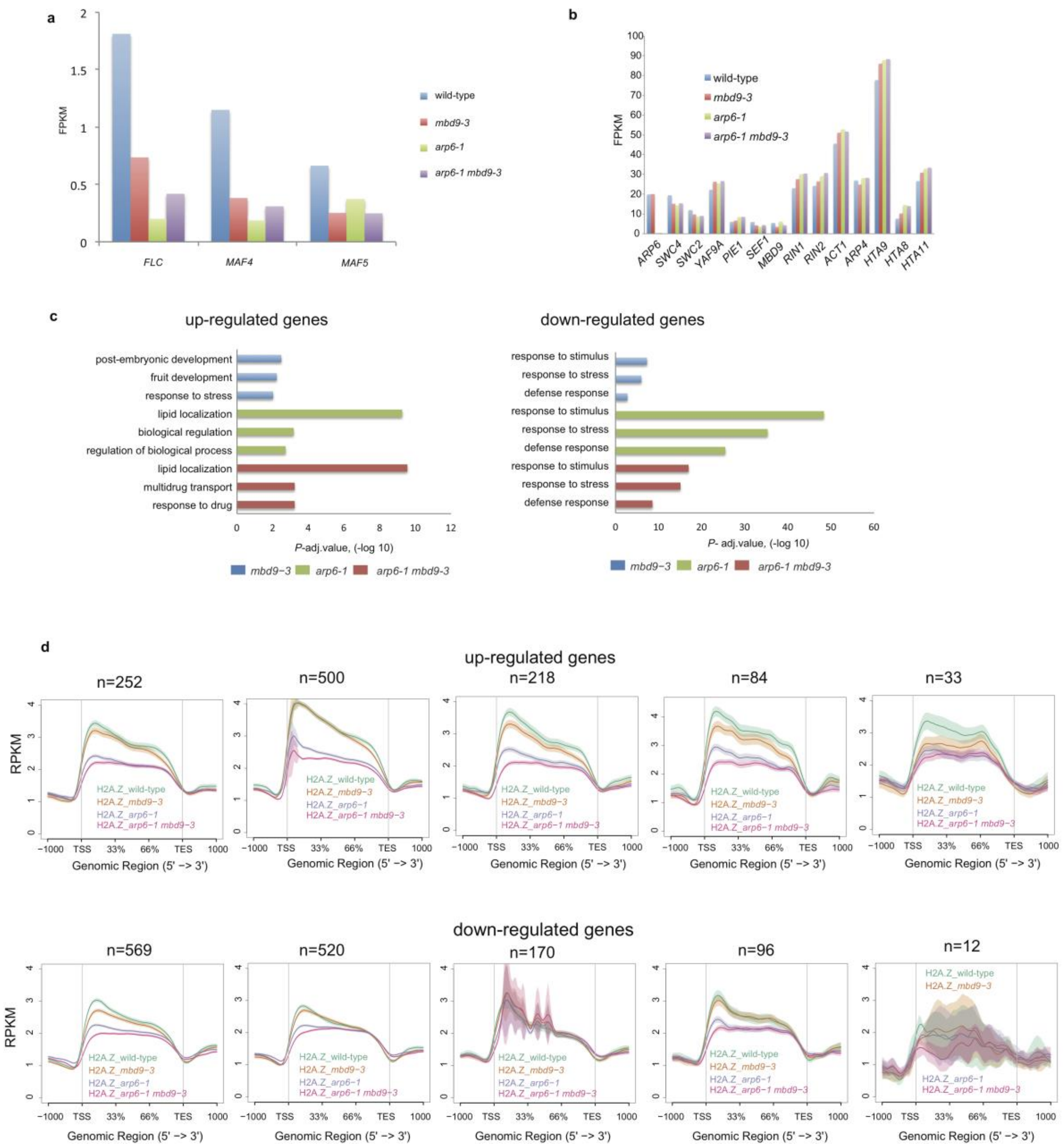

Supplementary Figure 5. Effects of the loss of H2A.Z on gene expression (a). Normalized expression levels (FPKM) of *FLC, MAF5,* and *MAF4* from RNA-seq (FPKM) in wild-type *mbd9-3, arp6-1*, and *arp6-1 mbd9-3* from four independent replicates for each sample. **(b)** Normalized expression levels (FPKM) of SWR1 complex components and H2A.Z genes in wild-type, *mbd9-3, arp6-1*, and *arp6-1 mbd9-3* from four independent replicates for each sample. **(c)** GO term analysis for significantly up-

regulated and down-regulated genes in each mutant vs wild-type control. *P*-adjusted value in –log(10) is shown for the top three GO classes for each mutant. **(d)** Distribution of normalized H2A.Z ChIP-seq signal (RPKM) from merged replicates over classes of overlapping up-regulated and down-regulated genes based on RNA-seq in wild-type, *mbd9-3, arp6-1*, and *arp6-1 mbd9-3*.

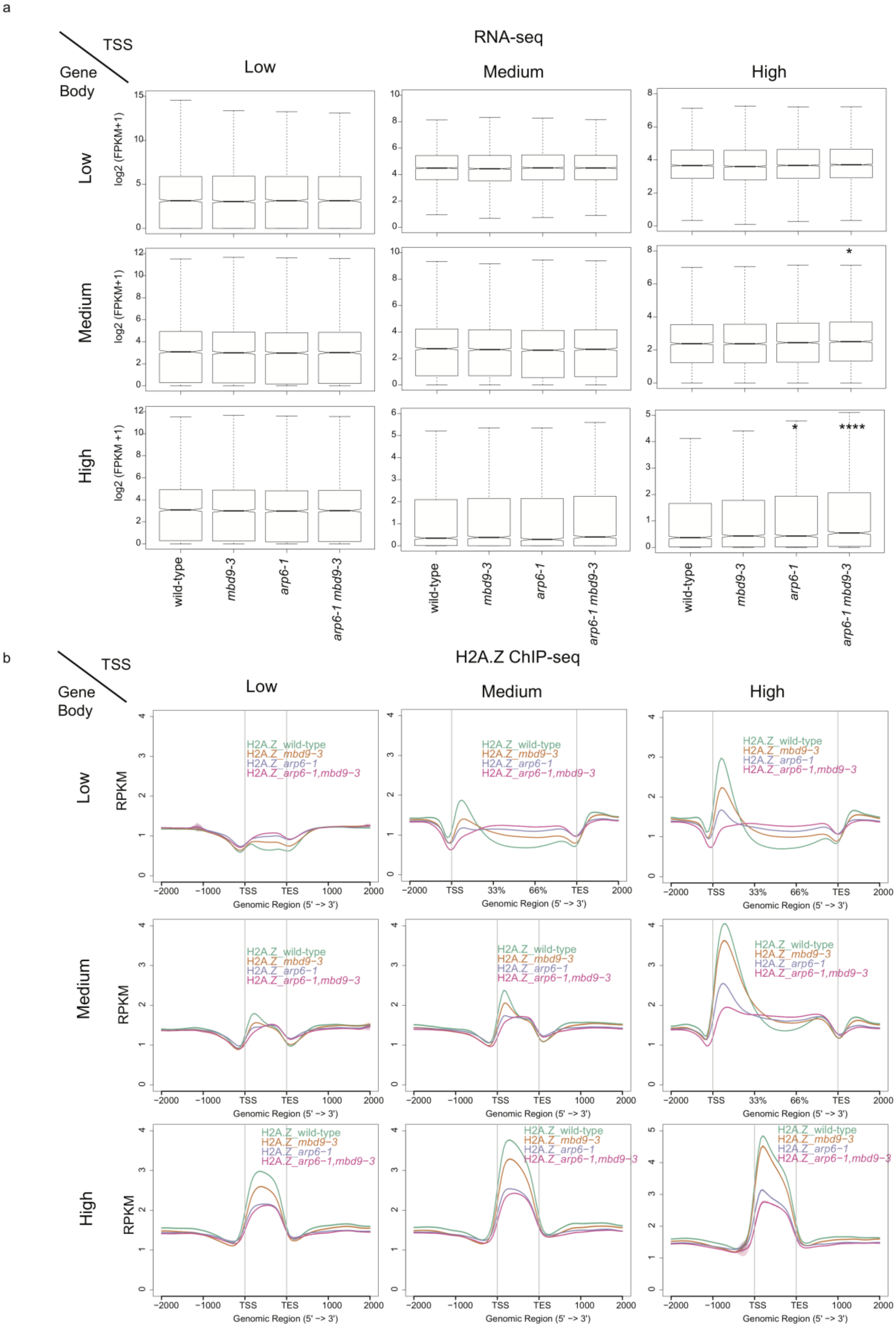
a
TSS
Gene Body
RNA-seq
Low
Medium
High
log2 (FPKM+1)
log2 (FPKM +1)
*
****
wild-type
mbd9-3
arp6-1
arp6-1 mbd9-3
b
TSS
Gene Body
H2A.Z ChIP-seq
Low
Medium
High
RPKM
H2A.Z_wild-type
H2A.Z_mbd9-3
H2A.Z_arp6-1
H2A.Z_arp6-1,mbd9-3
-2000
-1000
TSS
TES
1000
2000
33%
66%
Genomic Region (5' -> 3')

Supplementary Figure 6. Expression and H2A.Z profiles at nine classes of H2A.Z-occupied genes in wild-type, *mbd9-3, arp6-1*, and *arp6-1 mbd9-3*. **(a)** Notched boxplots of average normalized RNA-seq reads $\log_2$ (FPKM +1) for nine classes of H2A.Z genes according to Supplementary Fig. 4. Centre line indicates the median, upper and lower bounds represent the 75th and the 25th percentile respectively, whiskers indicate the minimum and the maximum, outliers are not plotted. Unpaired two-samples Wilcoxon test was used to determine significance between wild-type and mutants, only significant values are shown; * p-value <= 0.05, ** p-value <= 0.01, *** p-value <= 0.001, **** p-value <= 0.0001. **(b)** Distribution of normalized H2A.Z ChIP-seq signal (RPKM) from merged replicates over the nine classes of H2A.Z genes according to Supplementary Fig. 4. Source data is provided as Source Data file.

a Transcription factors up and down regulated in *mbd9-3*, *arp6-1* and *arp6-1 mbd9-3* mutants

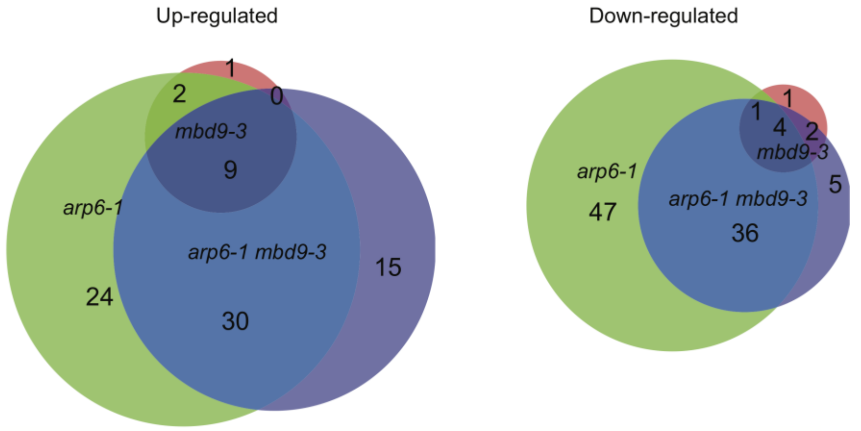

b Transcription factor families up-regulated in *mbd9-3*, *arp6-1* and *arp6-1 mbd9-3* mutants

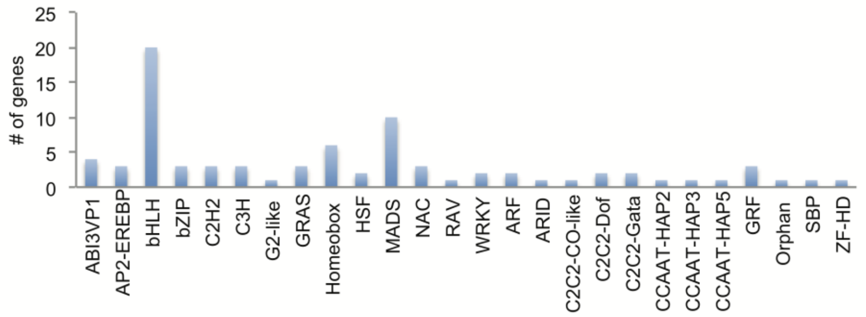

Transcription factor families down-regulated in *mbd9-3*, *arp6-1* and *arp6-1 mbd9-3* mutants

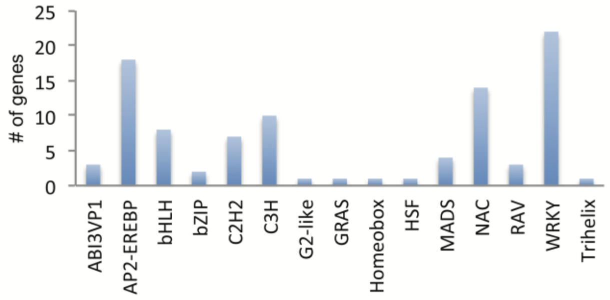

Supplementary Figure 7. Distribution of up-regulated and down-regulated transcription factors and transcription factor families in *mbd9-3, arp6-1*, and *arp6-1 mbd9-3* mutants. (a) Intersection of significantly up-regulated and down-regulated genes with transcription factors obtained from AtTFDB (agris-knowledge.com). **(b)** Distribution of transcription factor families obtained from AtTFDB (agris-knowledge.com) intersected with up-regulated and down-regulated genes.

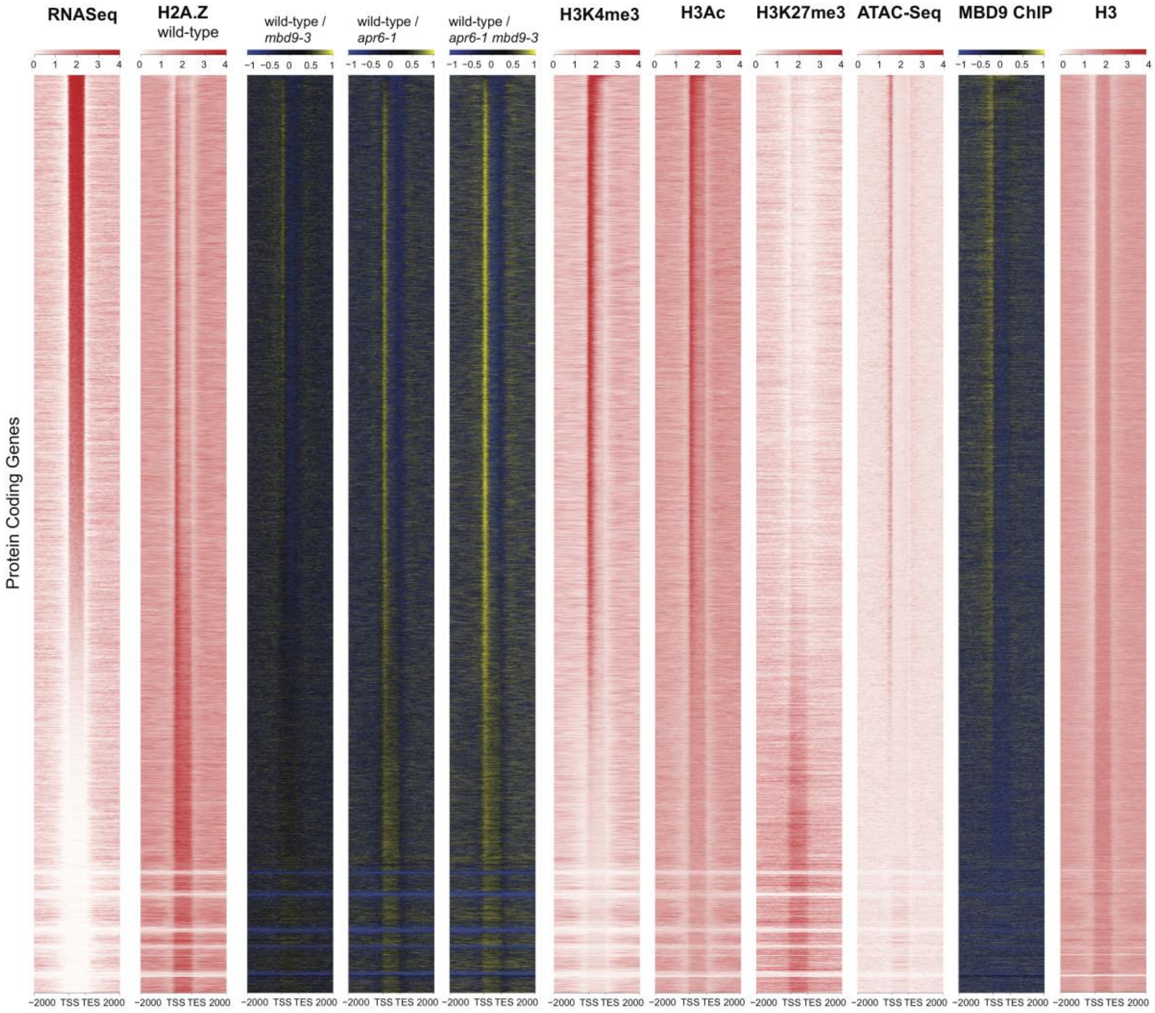

Supplementary Figure 8. Heatmap of normalized expression (RPKM) and histone modifications (RPKM) plotted in relation to decreasing levels of expression in wild-type for RNA-seq and ChIP-seq for H2A.Z in wild-type, ratio plots for H2A.Z vs wild-type for *mbd9-3, arp6-1, arp6-1 mbd9-3* mutants, H3K4me3, H3AC, H3K27me3, and H3 in wild-type, ATAC-seq in wild-type and ratio plot for MBD9-FLAG vs wild-type.

Supplementary Table 1. List of primers used in this study.

Primer Name	Sequence	Description
JP12457	CACCGTACATCACATGTTACATGCA	Forward primer ~1kb upstream of MBD9, with CACC tag added for Gateway Cloning into P-ENTR D TOPO (Life Technologies)
JP12458	GGATCCCTCGGGTTCTTTCCT	Reverse primer -stop codon for MBD9
AtARP6-F	CACCAGCCACAGGAGGAAAGAGAAAT	Forward primer ~1,5kb upstream of ARP6, with CACC tag added for Gateway Cloning into P-ENTR D TOPO (Life Technologies)
AtARP6-R	ATGAAAGAATCGTCTACGACACC	Reverse primer -stop codon for ARP6
JP3628	TTCTCCAAACGTCGCAACGGTCTC	FLC RT-PCR
JP3629	GATTTGTCCAGCAGGTGACATCTC	FLC RT-PCR
MAF4-F1	TCAAGTAACCACCATCACCAACG	MAF4 RT-PCR
MAF4-R1	CAAGAACACACAGAAAAGCACGAA	MAF4 RT-PCR
MAF5-F1	AGACAAAACTCAGGATTATCTTTCACAC	MAF5 RT-PCR
MAF5-R1	ACTTACATTATCCCCTTTTGCTTCTT	MAF5 RT-PCR
JP3483	GATCTTTGCCGGAAAACAATTGGAGG	UBQ10 RT-PCR
JP3484	CGACTTGTCATTAGAAAGAAAGAGAT	UBQ10 RT-PCR

Supplementary References

1 Langmead, B., Trapnell, C., Pop, M. & Salzberg, S. L. Ultrafast and memory-efficient alignment of short DNA sequences to the human genome. *Genome Biol* **10**, R25, (2009).

2 Li, H. *et al.* The Sequence Alignment/Map format and SAMtools. *Bioinformatics* **25**, 2078-2079, (2009).

3 Nix, D. A., Courdy, S. J. & Boucher, K. M. Empirical methods for controlling false positives and estimating confidence in ChIP-Seq peaks. *BMC Bioinformatics* **9**, 523, (2008).

4 Freese, N. H., Norris, D. C. & Loraine, A. E. Integrated genome browser: visual analytics platform for genomics. *Bioinformatics* **32**, 2089-2095, (2016).

5 Zhang, Y. *et al.* Model-based analysis of ChIP-Seq (MACS). *Genome Biol* **9**, R137, (2008).

6 Hulsen, T., de Vlieg, J. & Alkema, W. BioVenn - a web application for the comparison and visualization of biological lists using area-proportional Venn diagrams. *BMC Genomics* **9**, 488, (2008).

7 Shen, L., Shao, N., Liu, X. & Nestler, E. ngs.plot: Quick mining and visualization of next-generation sequencing data by integrating genomic databases. *BMC Genomics* **15**, 284, (2014).

8 Heinz, S. *et al.* Simple combinations of lineage-determining transcription factors prime cis-regulatory elements required for macrophage and B cell identities. *Mol Cell* **38**, 576-589, (2010).

9 Du, Z., Zhou, X., Ling, Y., Zhang, Z. & Su, Z. agriGO: a GO analysis toolkit for the agricultural community. *Nucleic Acids Res* **38**, W64-70, (2010).
10 Krueger, F. & Andrews, S. R. Bismark: a flexible aligner and methylation caller for Bisulfite-Seq applications. *Bioinformatics* **27**, 1571-1572, (2011).
11 Trapnell, C. *et al.* Transcript assembly and quantification by RNA-Seq reveals unannotated transcripts and isoform switching during cell differentiation. *Nat Biotechnol* **28**, 511-515, (2010).
12 Tian, T. *et al.* agriGO v2.0: a GO analysis toolkit for the agricultural community, 2017 update. *Nucleic Acids Res* **45**, W122-W129, (2017).
13 Coleman-Derr, D. & Zilberman, D. Deposition of histone variant H2A.Z within gene bodies regulates responsive genes. *PLoS Genet* **8**, e1002988, (2012).
14 Greenberg, M. V. *et al.* Interplay between active chromatin marks and RNA-directed DNA methylation in Arabidopsis thaliana. *PLoS Genet* **9**, e1003946, (2013).
15 Li, C. *et al.* The Arabidopsis SWI2/SNF2 chromatin Remodeler BRAHMA regulates polycomb function during vegetative development and directly activates the flowering repressor gene SVP. *PLoS Genet* **11**, e1004944, (2015).
16 Zhong, X. *et al.* DDR complex facilitates global association of RNA polymerase V to promoters and evolutionarily young transposons. *Nat Struct Mol Biol* **19**, 870-875, (2012).
